# Time-Series Multi-Omics Revealed Candidate Regulators of Antioxidant-Mediated Chilling Tolerance in Japonica Rice at Rice Booting Stage

**DOI:** 10.3390/antiox15101245

**Published:** 2026-09-27

**Authors:** Zhenhua Guo, Lijun Cai, Xirui Zhang, Xuesong Zhou, Yongsheng Cai, Hu Cao, Peng Li, Junxiang Guo, Wendong Ma, Yanjiang Feng

**Affiliations:** Rice Research Institute of Heilongjiang Academy of Agricultural Sciences, Jiamusi 154026, China

**Keywords:** japonica rice, booting stage, cold stress, antioxidant system, transcriptomics, metabolomics

## Abstract

Chilling damage at the booting stage represents a primary constraint limiting stable yields of japonica rice in cold-growing regions, yet the molecular mechanisms driving genotypic variation in chilling tolerance is still unclear. In this study, the cold-tolerant cultivar Kongyu 131 and the cold-sensitive cultivar Longjing 11 were used as contrasting materials and subjected to low-temperature treatment at the booting stage for 0 h (T1), 72 h (T3), and 120 h (T5). The results showed that low-temperature was the dominant factor driving the divergence in yield traits between the two varieties: after T5 treatment, the seed setting rate (SSR) and grain weight per plant (GW) of KY131 were 16.76% and 38.96% higher than those of LJ11, respectively. Physiologically, KY131 maintained higher SOD, POD, and CAT activities and proline content throughout the treatment, with significantly lower accumulation of O_2_^−^ and H_2_O_2_. Metabolomic profiling detected 101 differentially accumulated metabolites (DAMs) between cultivars, which were enriched in of fructose and mannose metabolism, amino sugar and nucleotide sugar metabolism, glycolysis/gluconeogenesis pathways. Notably, D-fructose 6-phosphate (Com_764_neg) was identified as a core DAM (VIP ≥ 1, *p* < 0.05) that accumulated to a greater extent in KY131 than in LJ11 under cold stress. Transcriptome analysis identified 692 differentially expressed genes (DEGs) between varieties, and integrated co-expression network and WGCNA analyses pinpointed *OsbZIP69* (Os08g0549600) as the candidate hub transcription factor with the highest connectivity, which showed the strongest co-expression association the hexokinase *OsHXK7* (Os05g0187100). The candidate *OsbZIP69*-*OsHXK7* association and the core genes identified in this study provided a theoretical basis and gene resources for molecular breeding of cold-tolerant japonica rice in cold regions.

## 1. Introduction

Rice (*Oryza sativa* L.) sustains over half of the global population, and consistent yield gains directly support global food security [1]. As rice cultivation expands into higher-latitude and high-altitude regions, chilling damage has become one of the major abiotic stresses threatening yield stability [2,3]. As China’s northernmost japonica rice production hub and vital premium grain base, Heilongjiang Province frequently encounters summer low-temperature shocks due to monsoon patterns. Recurrent sterility-type cold damage at booting and heading stages has consistently caused severe grain losses [4]. Integrated meteorological and agronomic data revealed critical cold injury thresholds of ~17 °C for cold-sensitive and ~16 °C for cold-tolerant varieties in Heilongjiang, and that the booting stage is most susceptible to low-temperature harm [5]. As climate change intensifies the frequency of extreme thermal events, mining cold-tolerant genetic resources and dissecting the molecular pathways governing cold tolerance in cold-climate japonica rice are highly practically relevant to securing rice yields across northern production zones.

The booting stage is the most cold-sensitive period in rice, coinciding with pollen mother cell meiosis and anther development [6,7]. Cytological and physiological studies have confirmed that low temperature disturbs the programmed degeneration of the anther tapetum, blocks nutrient supply to developing microspores, causes anther shrinkage and a reduced proportion of fertile pollen, and ultimately manifests as increased empty-grain percentage and decreased seed setting rate and yield [5,7,8]. From a metabolic standpoint, impaired carbohydrate transport drove cold-induced pollen abortion, wherein chilling suppressed the anther-specific invertase gene *OsINV4*, leading to sucrose accumulation, hexose depletion, and defective starch filling that culminated in pollen sterility, while cold-tolerant cultivars sustained stable *OsINV4* expression to preserve starch synthesis [9]. Consistent with this metabolic disruption, chilling downregulated anther monosaccharide transporters (e.g., *MST8*), perturbing sugar partitioning between the tapetum and microspores; furthermore, exogenous sucrose pretreatment partially rescued pollen fertility and seed set, thereby establishing a causal link between carbohydrate availability and pollen viability [10,11]. Thus, stable carbon metabolism in cold-stressed young panicles distinguishes cold-tolerant cultivars from susceptible lines.

Beyond disrupted carbon metabolism, oxidative stress represented another primary driver of cold damage. Low temperatures impaired bio-membranes, reducing lipid fluidity and integrity, and induced excessive reactive oxygen species (ROS) such as superoxide anion (O_2_^−^) and hydrogen peroxide (H_2_O_2_) causing oxidative damage to proteins, nucleic acids, and photosystem [12,13]. Plants employed integrated antioxidant defenses, wherein superoxide dismutase (SOD) converts O_2_^−^ to H_2_O_2_ for detoxification by peroxidase (POD) and catalase (CAT), while compatible solutes such as proline (Pro) enhanced tolerance through osmotic regulation and radical scavenging [14,15]. Importantly, ROS exhibited a dual functionality, acting as both cytotoxic metabolites and vital signaling molecules; moderate H_2_O_2_ accumulation triggers MAPK-mediated transcriptional activation of defense genes, defining cold tolerance not as absolute ROS scavenging, but as the precise maintenance of redox homeostasis that balances oxidative damage against signal transduction efficacy [16,17,18]. Pot experiments revealed that cold-tolerant rice cultivars exhibit superior SOD, POD, and CAT activities, alongside higher soluble sugar and protein levels, coupled with attenuated lipid peroxidation compared to sensitive lines under booting-stage chilling [19]. These findings establish antioxidant capacity as a pivotal physiological determinant underlying genotypic variation in cold tolerance.

At the molecular level, rice cold tolerance was coordinately regulated by multiple genes and pathways, and characterization of associated genes delivered actionable targets for cold-tolerance breeding [20]. During cold signal perception, the G-protein regulator *COLD1* interacted with the Gα subunit to trigger Ca^2+^ influx, forming an early cold-sensing molecular switch in rice [21]. Multiple quantitative trait loci (QTLs) governing booting-stage cold tolerance were mapped, and functional genes including *CTB1*, *CTB3*, and *CTB4a* were cloned; their mechanisms sustained anther gibberellin homeostasis and ROS scavenging [22,23,24,25,26]. Transcriptionally, diverse TF families drove cold-response cascades: miR319-targeted TCP TFs modulated leaf morphogenesis and cold performance [27]; *OsERF096* mediated cold responses through the jasmonic acid (JA) signaling [28]; several repressors modulated anther dehiscence and fertility through GA pathways [14]. Among them, the bZIP family acted as central mediators of ABA and abiotic stress signaling. The rice genome encoded 89 OsbZIP proteins, 26 of which were upregulated by dehydration, salinity or cold [29]. *OsbZIP72* enhanced cold tolerance at both seedling and booting stages by activating ROS-scavenging genes to maintain intracellular redox homeostasis [30]. Sugar signaling components represented an emerging focus in this field: hexokinase (HXK) functions as both a glycolytic gatekeeper and a glucose sensor regulating abiotic stress responses [31,32]. The rice cytosolic isoform *OsHXK7* integrated sugar signaling and metabolism to sustain energy production under hypoxia via enhanced glycolytic fermentation [33,34]. Notably, HXK function activity carried dual risks: overactivated HXK stimulated ROS synthesis via upregulating respiratory burst oxidase homologues (RBOHs) and impaired stress tolerance. In wheat, overexpression of *TaHXK7-1A* reduced drought tolerance in transgenic *Arabidopsis* and wheat via this pathway, whereas the upstream TF *TabHLH148-5A* preserved stress resistance by binding the E-box within the *TaHXK7-1A* promoter to repress its expression [35]. Nevertheless, few studies have explored how cold-tolerant rice transcription factors modulate *HXK7* activity under stress and coordinate its catalytic function with ROS homeostasis.

The integration of high-throughput omics offers a robust framework for dissecting complex stress-response networks. RNA-seq revealed dynamic transcriptional reprogramming during cold responses [36], whereas widely targeted metabolomics resolved stress-induced metabolic profiles [37]. Combined multi-omics strategies reconstructed rice metabolic networks across developmental stages [38] and uncovered transcriptional-metabolic cascades governing brown planthopper resistance [39]. Weighted gene co-expression network analysis (WGCNA) further streamlined candidate gene mining by grouping genes into trait-linked modules and detecting intramodular hub genes, and was extensively adopted in crop stress research [40,41]. However, integrative multi-omics studies identifying core cold-tolerance regulators in cold-region japonica rice at the booting stage remained scarce.

In this study, a single cold-tolerant cultivar (Kongyu 131) was compared with a single cold-sensitive cultivar (Longjing 11) under booting-stage cold stress. To our knowledge, this represents the first integrated physiological, metabolomic, and transcriptomic analysis of booting-stage cold tolerance in japonica rice from Hei-longjiang Province. Following low-temperature treatment at the booting stage, we integrated agronomic trait assessments, antioxidant physiology of young panicles, widely targeted metabolomics, and RNA-seq data to construct gene co-expression networks via WGCNA. Our objectives were threefold: (1) to characterize differential agronomic and antioxidant responses to cold; (2) to identify key metabolites, differentially expressed genes (DEGs), and enriched pathways governing cold tolerance; and (3) to pinpoint core modules and hub transcription factors related to cold tolerance. This study aims to provide a theoretical framework and novel genetic resources for dissecting cold-tolerance mechanisms and advancing molecular design breeding in cold-region rice.

## 2. Materials and Methods

### 2.1. Plant Materials and Cultivation

Two cold-region japonica rice varieties with similar growth durations were used: the cold-tolerant cultivar Kongyu 131 (KY131, V1) and the cold-sensitive cultivar Longjing 11 (LJ11, V2); seeds were provided by the Rice Research Institute of Heilongjiang Academy of Agricultural Sciences. Their matched growth schedules and genetic backgrounds made them ideal for comparative cold tolerance research.

The experiment was conducted by pot cultivation in an artificial climate chamber [42], following a randomized complete block design with the pot as the experimental unit. Sowing was performed in April, and three-leaf seedlings were transplanted into plastic pots (30 cm in diameter and 25 cm in height) filled with approximately 12 kg of air-dried loam soil, with nine plants per pot and 30 pots per cultivar; the 30 pots of each cultivar were randomly allocated to three treatment groups (10 pots per treatment), and pots of the two cultivars were interleaved (randomized) within the chamber. Field management followed the standard cultivation practice for japonica rice in Heilongjiang Province: urea, diammonium phosphate, and potassium sulfate were applied at a ratio of 2:1:1, providing a total of 120 kg N·ha^−1^, 60 kg P_2_O_5_·ha^−1^, and 60 kg K_2_O·ha^−1^ over the whole growth period; a standing water layer of 3–5 cm was maintained in the pots throughout the growth period. At the booting stage, uniformly growing plants with panicles of 2–3 cm (early booting stage) were tagged and the tagged plants transferred to the artificial climate chamber for low-temperature treatment at 12 °C for three durations: 0 h (T1), 72 h (T3), and 120 h (T5), with 10 pots per treatment. During cold treatment, plants were illuminated by white LED panels providing a photosynthetic photon flux density (PPFD) of approximately 222 µmol·m^−2^·s^−1^ at the canopy level (i.e., 12,000 lx), with day/night relative humidity of 80%/70%, under a 16 h light/8 h dark photoperiod. Control plants were maintained under normal conditions (28 °C/22 °C day/night temperature, 60–70% relative humidity, and a 16 h light/8 h dark photoperiod) in a parallel growth chamber throughout the experiment. After cold treatment, plants were returned to normal conditions and grown to maturity, and panicle length, seed setting rate, and grain weight per plant were measured on three plants per pot at maturity.

### 2.2. Measurement of Antioxidant Physiological Indices

Young panicles were sampled at 0, 72, and 120 h of cold treatment: three uniformly growing plants were randomly selected per treatment, three young panicles were collected from each plant, and three biological replicates were set; samples were flash-frozen in liquid nitrogen and stored at −80 °C until use. Superoxide dismutase (SOD) activity was determined by the nitroblue tetrazolium (NBT) photoreduction method [11]; peroxidase (POD) activity was assayed by the guaiacol method [43]; catalase (CAT) activity was measured by the ultraviolet absorption method [44]; superoxide anion (O_2_^−^) content was determined following Huang et al. [45]; malondialdehyde (MDA) and Pro contents were measured by the thiobarbituric acid colorimetric method and the acid ninhydrin method, respectively [46]; and hydrogen peroxide (H_2_O_2_) content was measured using a commercial kit [47].

### 2.3. Widely Targeted Metabolomic Analysis

Metabolomic samples were collected in parallel with those in Section 2.2 (three time points, three biological replicates per time point), flash-frozen in liquid nitrogen, stored at −80 °C, and shipped on dry ice to a professional biotechnology company (Genedenovo Biotechnology Co., Ltd., Guangzhou, China) for analysis [48]. Sample pretreatment was as follows: freeze-dried young panicles were thoroughly ground using a grinder (MM400, Retsch GmbH, Haan, North Rhine-Westphalia, Germany); approximately 100 mg of powder was precisely weighed and resuspended in 500 μL of pre-chilled 80% methanol aqueous solution by thorough vortexing; the samples were incubated on ice for 5 min and centrifuged at 15,000× *g* and 4 °C for 20 min; an aliquot of the supernatant was diluted with LC-MS-grade water to a final methanol content of 53% and centrifuged again at 15,000× *g* and 4 °C for 20 min, and the resulting supernatant was filtered through a 0.22 µm hydrophilic microporous membrane (nylon; SCAA-104, ANPEL, Shanghai, China) before LC-MS/MS analysis. A pooled quality-control (QC) sample was prepared by mixing equal aliquots of all sample extracts and was used to equilibrate the chromatography–mass spectrometry system and to monitor instrument stability throughout the analytical run; in parallel, blank samples were included to remove background ions.

LC-MS/MS analysis was performed on an ExionLC™ AD UPLC system coupled to a QTRAP^®^ 6500+ triple-quadrupole linear-ion-trap mass spectrometer (AB SCIEX, Framingham, MA, USA). Chromatographic separation was achieved on a Waters Xselect HSS T3 column (2.1 mm × 150 mm, 2.5 μm) maintained at 50 °C, with a flow rate of 0.4 mL·min^−1^. The mobile phases consisted of 0.1% formic acid in water (eluent A) and 0.1% formic acid in acetonitrile (eluent B), with the following 20-min gradient program: 2% B at 0–2 min; 2–100% B at 2–15 min; 100% B at 15–17 min; 100–2% B at 17.1 min; and 2% B at 17.1–20 min for re-equilibration. The mass spectrometer was equipped with an electrospray ionization (ESI) source and operated in both positive and negative ion modes with the following parameters: curtain gas, 35 psi; collision gas, medium; ion-spray voltage, +5500 V (positive) and −4500 V (negative); source temperature, 550 °C; ion source gas 1, 60 psi; ion source gas 2, 60 psi. Metabolite detection was performed in multiple reaction monitoring (MRM) mode based on the Genedenovo in-house database: the Q3 (product ion) traces were used for metabolite quantification, whereas the Q1 (precursor ion), Q3, retention time (RT), declustering potential (DP), and collision energy (CE) were used for metabolite identification. The raw data files were processed with SCIEX OS software (version 1.4, AB SCIEX, Framingham, MA, USA) for chromatographic peak integration and correction, with the main parameters set as follows: minimum peak height, 500; signal-to-noise ratio, 5; Gaussian smoothing width, 1 point. The peak area of each chromatographic peak represented the relative content of the corresponding metabolite.

Quality control was implemented as follows: a pooled QC sample prepared by mixing equal aliquots of all extracts was injected every ten analytical runs to monitor instrument stability; extraction blanks were included; sample injection order was randomized; metabolites with a relative standard deviation (RSD) > 30% in QC samples were excluded; missing values were imputed with half of the minimum value, and data were log_2_-transformed and Pareto-scaled before multivariate analysis. Metabolites were annotated against the Metware database (MWDB) and public databases using retention time and MRM transition information, with identification confidence levels assigned according to the Metabolomics Standards Initiative. Moreover, metabolite identification was based on matching against a local database and public databases including MassBank, KNApSAcK, HMDB [49], MoTo DB, and METLIN [50]. The reliability of the data was evaluated by principal component analysis (PCA) of all samples including the QC samples, in which the tight clustering of the QC samples indicated satisfactory stability of the analytical system. Differentially accumulated metabolites (DAMs) were screened using variable importance in projection (VIP) ≥ 1 in the OPLS-DA model and *p* < 0.05 in the *t*-test. A permutation test was applied to evaluate OPLS-DA model accuracy: the class labels (Y) were randomly permuted 200 times, generating the permuted statistics R^2^′and Q^2^; the model was considered reliable and free of overfitting when all permuted Q^2^ values were lower than the Q^2^ value of the original model and the regression line of Q^2^ intersected the vertical axis at or below zero (Q^2^ intercept ≤ 0). Pathway annotation and enrichment analysis of DAMs were performed based on the KEGG database (http://www.kegg.jp/kegg/pathway.html, accessed on 15 September 2026).

### 2.4. Transcriptome Sequencing and Differential Expression Analysis

Meanwhile, fresh young panicles were collected from three plants per treatment (three biological replicates, sampled independently of the metabolomic samples to avoid cross-contamination), flash-frozen in liquid nitrogen, and submitted for total RNA extraction and transcriptome sequencing. Total RNA was extracted using TRIzol^®^ reagent (Invitrogen Corporation, Carlsbad, CA, USA), and its concentration, purity, and integrity were examined by an Agilent 2100 Bioanalyzer (Agilent Technologies Inc., Santa Clara, CA, USA) and agarose gel electrophoresis. Only RNA samples meeting the following quality criteria were used for library construction: A260/A280 ratio of 1.9–2.1 and A260/A230 ratio ≥ 2.0, RNA integrity number (RIN) ≥ 8.0 with a 28S/18S ratio ≥ 1.5. Qualified samples were used to construct cDNA libraries using the NEBNext^®^ Ultra™ RNA Library Prep Kit for Illumina^®^ (NEB #E7530, New England Biolabs, Ipswich, MA, USA) following standard protocols and sequenced on the Illumina HiSeq 2500 platform. After quality control, clean sequencing reads were mapped to the japonica rice *Nipponbare* reference genome (IRGSP-1.0) using HISAT2 (version 2.2.1, https://daehwankimlab.github.io/hisat2, accessed on 15 September 2026) [51]. Gene read counts were calculated with FeatureCounts, and gene expression levels were quantified as FPKM values.

Differential expression analysis was performed using DESeq2, with multiple testing corrected by the false discovery rate (FDR); genes satisfying |log_2_FC| > 1 and FDR < 0.05 were defined as differentially expressed genes (DEGs). GO functional annotation and KEGG pathway enrichment analysis of DEGs were conducted using the OmicShare online platform (https://www.omicshare.com/tools, accessed on 15 September 2026) [52]. Furthermore, Mfuzz soft clustering and hierarchical clustering were conducted on DEGs based on FPKM values to characterize dynamic gene expression patterns, and a transcription factor–target gene co-expression network was subsequently constructed. The TF-target gene network was constructed by motif-based TFBS prediction using eight TFs and 25 associated DEGs from Mfuzz temporal clustering as input. PFMs of TF binding motifs were obtained from JASPAR 2026 by matching rice TF protein sequences to JASPAR-annotated plant homologs. Promoter sequences (1000 bp upstream of the TSS) of candidate target genes were extracted from the *Nipponbare* reference genome (IRGSP-1.0). TF motifs were scanned against these promoters using FIMO (MEME Suite, version 5.5.4) with a *p*-value threshold of 1 × 10^−4^; genes with at least one significant motif occurrence were retained as putative targets. TF-target pairs were assembled into a regulatory network, visualized in Cytoscape 3.9.1, and node degree was used to prioritize hub TFs.

### 2.5. Co-Expression Network Construction and Hub Gene Mining

Using the up-regulated DEGs at each time point as input data, low-expression genes (FPKM < 1) were removed, and a weighted gene co-expression network was constructed with the R package WGCNA (v1.69) [40]: samples were first hierarchically clustered to exclude outliers, and the soft-thresholding power β = 14 was selected as the lowest power at which the scale-free topology fit index reached R^2^ ≥ 0.85; a signed network was constructed with a minimum module size of 30 and a module-merging cut height of 0.25, yielding 16 co-expression modules. Subsequently, Pearson correlation coefficients between module eigengenes and antioxidant physiological indices as well as DAMs were calculated to identify key modules significantly associated with target traits, with *p* values adjusted by the Benjamini–Hochberg procedure across all module–trait tests; hub genes were defined by module membership |MM| ≥ 0.8 and gene significance |GS| ≥ 0.2. The genes within key modules were subjected to GO and KEGG enrichment analyses using the OmicShare platform [52]. The core transcription factors obtained from the TF–target network were intersected with the hub transcription factors within WGCNA key modules, and the intersecting members were defined as candidate cold-tolerance hub genes. Finally, based on gene–gene co-expression regulatory relationships, a candidate gene interaction network was drawn using Cytoscape 3.9.1 [53].

### 2.6. Data Analysis

Agronomic trait and physiological-biochemical data were compiled in Microsoft Excel 2021 and graphed with GraphPad Prism 9.0. Significance of differences among treatments was evaluated by *t*-test and one-way analysis of variance (one-way ANOVA), followed by the LSD multiple comparison test using Statistix 8.0. Data were analyzed by two-way analysis of variance (two-way ANOVA) with cultivar, cold duration, and their interaction (cultivar × duration) as fixed effects, followed by Tukey’s HSD post-hoc tests; *F* statistics, degrees of freedom, exact *p* values, effect sizes (partial η^2^), and 95% confidence intervals for key contrasts are reported (Table S1); figures were produced with GraphPad Prism 10 [48]. Different lowercase letters in the figures indicate significant differences among treatments (*p* < 0.05), while * and ** denote significance at *p* < 0.05 and *p* < 0.01, respectively. Multivariate statistical analyses, including PCA, VIP, and correlation analyses, were performed in the R environment.

## 3. Results

### 3.1. Effects of Cold Stress at the Booting Stage on Rice Phenotype and Agronomic Traits

Yield component analysis showed that cold treatment directly affected agronomic traits at the booting stage (Figure 1A–D). Under T1 condition, the seed setting rate (SSR, *p* < 0.05) and grain weight per plant (GW, *p* < 0.01) of KY131 was significantly higher than that of LJ11, whereas its panicle length (PL) was notably shorter than that of LJ11, indicating that under normal conditions KY131 achieved significantly higher yield per plant through a higher seed setting level (Figure 1E–G). Two-way ANOVA (Table S1) confirmed highly significant cultivar × duration interactions for both SSR (F(1, 16) = 12.07, *p* = 0.003, partial η^2^ = 0.43) and GW (F(1, 16) = 69.18, *p* < 0.0001, partial η^2^ = 0.81), demonstrating that KY131 and LJ11 responded differently to prolonged cold rather than merely differing constitutively. After T5 treatment, PL decreased in both varieties with no significant difference between them; however, the varietal differences in SSR and GW were further amplified: the SSR of KY131 was significantly higher (*p* < 0.01) than LJ11 with an increase of 16.8%; the GW of KY131 was significantly higher than LJ11 (*p* < 0.01), an increase of 39.0%. In terms of within-cultivar reduction, cold treatment decreased the SSR of KY131 by 10.6%, whereas that of LJ11 dropped by as much as 20.1%, meanwhile the GW decreased sharply in both varieties under cold stress (Figure 1E–G). These results indicated that low temperature was the main driver further widening the differences in SSR and GW between the two varieties, and that KY131 possessed significantly stronger cold tolerance than LJ11 at the booting stage, making them an ideal contrasting pair for dissecting the cold-tolerance mechanism of cold-region japonica rice at the booting stage.

### 3.2. Effects of Cold Stress on the Antioxidant System

Measurements of the antioxidant defense system (Figure 2) revealed that KY131 effectively suppressed ROS accumulation under chilling stress by sustaining higher antioxidant enzyme activities. SOD activity in young panicles was consistently higher in KY131 than in LJ11 across all chilling treatments (Figure 2A). POD activity did not differ significantly between the two cultivars at T1, but was markedly higher in KY131 than in LJ11 at T3 and T5 (*p* < 0.01, Figure 2B). CAT activity was highly significantly elevated in KY131 relative to LJ11 at all time points (*p* < 0.01, Figure 2C). Notably, proline (Pro) content was also higher in KY131 than in LJ11 at both the T1 and T5 time points (Figure 2D). The two-way ANOVA showed significant cultivar main effects for SOD (F(1, 12) = 23.85, *p* = 0.0004), POD (F(1, 12) = 22.98, *p* = 0.0004), and CAT (F(1, 12) = 276.84, *p* < 0.0001) with non-significant cultivar × duration interactions, indicating that the superior antioxidant capacity of KY131 was largely constitutive; Pro content did not differ significantly between the two cultivars.

Corresponding to the varietal differences in antioxidant enzyme activities, ROS contents showed an opposite accumulation trend. As shown in Figure 2E, the O_2_^−^ content of KY131 was significantly lower than that of LJ11 at T1 (*p* < 0.05); in terms of within-cultivar changes, cold treatment significantly increased the O_2_^−^ content of KY131 by 3.0% (*p* < 0.05), whereas that of LJ11 remained essentially unchanged, but the absolute level of KY131 was still lower than that of LJ11. As shown in Figure 2G, the H_2_O_2_ content of KY131 was lower than that of LJ11 at all time points. MDA contents did not differ significantly between the two varieties at any time point (two-way ANOVA: cultivar F(1, 12) = 0.29, *p* = 0.599; interaction *p* = 0.416; Figure 2F).

### 3.3. Multivariate Statistical Analysis of Agronomic Traits and Antioxidant Indices

To comprehensively resolve the association patterns among agronomic traits and antioxidant indices of the two varieties under cold treatment, the PCA, VIP, and correlation analysis were performed on the ten indices across four treatment groups (V1T1, V1T5, V2T1, V2T5) (Figure 3). The PCA results (Figure 3A,B) showed that the first principal component (PC1) explained 94.10% of the variance and the second (PC2) explained 5.50%; the four treatment groups were clearly separated on the score plot Loading analysis showed that O_2_^−^ pointed toward the LJ11 side, whereas SOD pointed toward the KY131 side, indicating that the overall varietal differences in antioxidant far exceeded the effect of treatment duration.

VIP analysis (Figure 3D) further screened the key indicators discriminating the two varieties: SOD activity showed the highest VIP value among the ten measured indicators, suggesting that it contributed most strongly to the separation of the two cultivars. Correlation analysis (Figure 3C,E) showed that SOD, POD, and CAT activities were significantly positively correlated with Pro content, forming a coordinated antioxidant module; SSR and GW were strongly positively correlated with each other, and both were positively correlated with SOD, CAT, and Pro but negatively correlated with O_2_^−^ and H_2_O_2_; panicle length was negatively correlated with POD, Pro, and CAT. These results confirmed from a multivariate statistical perspective that antioxidant capacity was the key physiological basis determining the stability of yield traits under booting-stage cold stress.

### 3.4. Effects of Cold Treatment on the Rice Metabolome

To explore the chemical basis underlying the difference in cold-tolerance between the two varieties, we used widely targeted metabolomics to systematically profile metabolites in young panicles under cold treatment. High data quality was confirmed by correlation analysis, with all samples showing R values > 0.88 and tight clustering of the three biological replicates per treatment (Figure 4A). A total of 101 DAMs (*p* < 0.05) were identified between the two varieties (Figure 4B,C, Table S1), while 30, 28, and 43 DAMs were identified in V2_T1 vs. V1_T1, V2_T3 vs. V1_T3, and V2_T5 vs. V1_T5.

KEGG pathway enrichment analysis (Figure 4D) showed that the DAMs were significantly enriched in several metabolic pathways, including fructose and mannose metabolism, amino sugar and nucleotide sugar metabolism, starch and sucrose metabolism, galactose metabolism, and arginine and proline metabolism, with sugar metabolism and amino acid metabolism pathways being predominant. Pearson correlation network analysis of the 15 core metabolites (Figure 4E, Table S2) suggested coordinated changes among these metabolites during the cold response. Further Sankey diagram analysis of pathways and metabolites (Figure 4F) showed that five pathways (i.e., fructose and mannose metabolism, amino sugar and nucleotide sugar metabolism, glycolysis/gluconeogenesis, arginine and proline metabolism, and shikimate-pathway alkaloid biosynthesis) converged on seven core metabolites, which were retained from the 15 core DAMs by satisfying both (i) VIP ≥ 1 and *p* < 0.05 in the pairwise cultivar comparisons and (ii) annotation to the KEGG pathways significantly enriched in this study. The heatmap of relative contents of these seven core metabolites across the six treatment groups (Figure 4G) revealed a distinct cultivar-specific accumulation pattern: the contents of F6P, D-glucose, shikimic acid, L-proline, and D-proline were generally higher in KY131 than in LJ11, and these differences became more pronounced with prolonged cold treatment.

### 3.5. Effects of Cold Treatment on the Rice Transcriptome

To investigate cold tolerance-related gene expression differences under cold stress, RNA-seq transcriptome sequencing and comparative analysis were performed on 18 young panicle samples of the two varieties at three time points. RNA-seq library sequencing and stringent quality filtering were performed across all 18 samples. In total, the raw sequencing data amounted to 1.12 × 10^11^ bp, and high-quality clean data after filtering reached 1.08 × 10^11^ bp. The average Q20, Q30, and GC content of clean reads were 97.47%, 93.41%, and 48.39%, respectively, demonstrating high sequencing accuracy and qualified transcriptomic data for subsequent bioinformatic analyses (Table S3). Principal component analysis (PCA) based on the expression levels of all detected genes showed (Figure 5A) that PC1 and PC2 explained 71.5% and 19.4% of the variance, respectively; the three biological replicates of each treatment clustered tightly, whereas samples of different varieties and treatment durations were clearly separated, indicating reliable sequencing data with good reproducibility. DEGs between the two varieties at each time point were identified by DESeq2 (|log_2_FC| > 1, FDR < 0.05) using LJ11 as the control. As shown in Figure 5B,C, 2179, 11,207, and 3116 DEGs were identified in V2_T1 vs. V1_T1, V2_T3 vs. V1_T3, and V2_T5 vs. V1_T5, respectively; transcriptional reprogramming was most dramatic at T3, indicating that 72 h of cold treatment is the period of strongest transcriptional divergence between the two varieties. Venn analysis showed that 692 DEGs were stably differentially expressed across all three time-point comparisons (Figure 5C, Table S4), constituting the core transcriptional response gene set underlying the cold-tolerance difference. KEGG enrichment analysis (Figure 5D) showed that these 692 common DEGs were significantly enriched in cutin, suberine and wax biosynthesis, diterpenoid biosynthesis, plant-pathogen interaction, fatty acid metabolism, galactose metabolism.

Mfuzz temporal clustering based on FPKM divided the 692 common DEGs into 10 expression clusters (Figure 5E, Table S5), which displayed distinct time- and cultivar-specific expression patterns and showed enrichment in different pathways. Among them, cluster 3, 4, 5, 7, and 8 were significantly enriched in KY131 after cold treatment, suggesting that genes in these clusters were predominantly up-regulated in this variety. A TF-target gene co-expression network was constructed based on the common DEGs (Figure 5F, Table S6), revealing eight transcription factors (*OsTDF1*, *OsERF128*, *OsERF33*, *EAT1*, *CM1*, *OsSTA280*, *PAS2A*, and *OsbZIP69*) and 25 target genes. Among them, *OsbZIP69* (Os08g0549600), *OsERF33* (Os04g0549700), and *OsTDF1* (Os03g0296000) had the highest connectivity and served as hub TFs; meanwhile, the hexokinase gene *OsHXK7* (Os05g0187100) appeared in the network as a target gene with high-score connections to *OsbZIP69*. KEGG enrichment analysis of the core network genes (Figure 5G) showed significant enrichment in galactose metabolism, amino sugar and nucleotide sugar metabolism, pentose and glucuronate interconversions, glycosphingolipid biosynthesis, cutin/suberine/wax biosynthesis.

### 3.6. Integrated Transcriptome-Metabolome WGCNA Mining of Core Cold-Tolerance Genes

To further dissect the regulatory mechanisms linking DAM changes to the DEG co-expression network, WGCNA was performed based on the up-regulated DEGs at each cold-treatment time point (Table S7). The co-expression module clustering dendrogram (Figure 6A) partitioned all genes into 16 co-expression modules. Module-trait correlation analysis (Figure 6B) showed that gene expression in multiple modules was significantly correlated with the seven core DAMs and antioxidant indices. The heatmap of module-trait correlations showed that gene expression in 4 core modules (black, magenta, green, pink) was significantly correlated with F6P, SOD activity, and O_2_^−^ content (Figure 6B, Table S8). KEGG circular enrichment analysis of genes in the ore modules (Figure 6C) identified amino sugar and nucleotide sugar metabolism (ko00520), glycolysis/gluconeogenesis (ko00010), and starch and sucrose metabolism (ko00500) as the core enriched pathways, confirming the central position of sugar metabolism pathways at the module level.

Intersection of the 13 core TFs screened from the DEG co-expression network with the 37 hub TFs from the WGCNA key modules (Figure 6D) yielded three overlapping core TFs. The co-expression network constructed from these overlapping core factors (Figure 6E, Table S9) revealed that *OsbZIP69* had the highest connectivity as a candidate hub factor, being co-expressed with 14 target genes, including the hexokinase gene *OsHXK7* (Os05g0187100), *OsSTA280*, *OsPME5*. KEGG enrichment analysis of the hub genes (Figure 6F) further confirmed that amino sugar and nucleotide sugar metabolism and starch and sucrose metabolism were the most significantly enriched pathways. Integrating the above results, a hypothetical working model for cold tolerance in cold-region japonica rice at the booting stage was constructed (Figure 6G).

## 4. Discussion

Combining agronomic phenotyping, antioxidant physiological assays, and integrated transcriptomic and metabolomic analyses, this study systematically dissected the molecular regulatory network governing booting-stage cold tolerance in cold-region japonica rice. We validated the essential roles of antioxidant homeostasis and sugar metabolism (particularly F6P) in cold tolerance and identified the candidate hub TF *OsbZIP69* and its putative target *OsHXK7* through WGCNA. Notably, previous hexokinase studies have focused largely on drought, salinity, or pathogen stress; evidence linking a specific hexokinase to booting-stage chilling tolerance in rice remains limited, and the association of *OsHXK7* expression with F6P accumulation and antioxidant homeostasis has not been reported previously. Referencing the reported mechanism by which wheat glucose sensor *TaHXK7-1A* promotes ROS accumulation and impairs drought resistance [35], we proposed a novel cold-tolerance regulatory model that explains the cold-adaptation mechanism of tolerant rice cultivars. These findings provide new theoretical insights and genetic resources for mechanistic cold-tolerance research and molecular breeding of northern japonica rice.

### 4.1. Effects of Low Temperature at the Booting Stage on Rice Agronomic Traits

Phenotypic analysis revealed that despite a shared genetic background, KY131 and LJ11 exhibited divergent agronomic responses to booting-stage chilling. Under normal conditions, KY131 displayed a significantly higher seed-setting rate and grain weight per plant but shorter panicles. Cold stress (120 h) exacerbated these differences: KY131 retained a 16.8% and 39.0% advantage in seed-setting rate and grain weight over LJ11, respectively, with a markedly smaller reduction in seed-setting rate. The stability of seed setting rate and grain weight in KY131 under cold stress reflected stronger protection of reproductive growth. These findings aligned with reports linking chilling-induced anther shrinkage and reduced fertile pollen to lower seed set across rice varieties [8], and mirror observations in Heilongjiang cultivars where cold-tolerant KY 131 exhibited minimal increases in spikelet sterility compared to sensitive lines [5]. Notably, the absence of varietal differences in panicle length post-stress suggested KY131 prioritizes assimilate allocation to reproductive sinks (grain filling) over vegetative growth (rachis elongation).

### 4.2. The Central Role of the Antioxidant System in Booting-Stage Cold Tolerance of Cold-Region Rice

Chilling-induced ROS accumulation acted as a primary driver of cellular damage, yet the antioxidant system performed roles extending beyond mere scavenging to coordinate systemic cold responses through signaling cascades [18]. In this study, the activities of CAT, SOD, and POD in young panicles of KY131 were all higher than those of LJ11. This enhanced enzymatic performance suppressed O_2_^−^ and H_2_O_2_ accumulation precluding irreversible oxidative damage [12,54]. Multivariate analyses supported these findings: PCA distinctly separated the two genotypes along PC1 (94.1% variance explained), while VIP analysis identified SOD activity as the most critical indicator discriminating the two varieties, and correlation analysis showed that SSR and GW were significantly positively correlated with SOD, CAT, POD, and Pro but negatively correlated with O_2_^−^ and H_2_O_2_. Collectively, these data identified the SOD-centric antioxidant enzyme system as the physiological foundation of booting-stage cold tolerance in cold-region japonica rice, aligning with previous reports comparing Longdao 5 and Longjing 11 [55]. Consistent with the non-significant differences in Pro and MDA contents between cultivars (Section 3.2), the comparable Pro and MDA content between cultivars indicated that the disparate cold tolerance observed herein was attributable more to variations in ROS-scavenging capability [56]. Furthermore, net O^2−^ accumulation changed modestly in both cultivars during cold treatment, consistent with rapid SOD-mediated dismutation.

### 4.3. Regulation of Rice Cold Tolerance by Sugar and Proline Metabolism

Metabolomic profiling revealed the biochemical basis of cold tolerance in KY131. Among 101 DAMs, KY131 preferentially accumulated soluble phosphorylated hexoses (e.g., F6P, glucose-6-phosphate). These metabolites primarily converged on fructose and mannose metabolism, amino sugar and nucleotide sugar metabolism, starch and sucrose metabolism, galactose metabolism, and arginine and proline metabolism. Soluble sugars, including sucrose, hexoses, raffinose, and trehalose, served multiple roles in cold defense: they provided energy and carbon skeletons, stabilized cellular membranes, and functioned as osmotic regulators and signaling molecules [57]. Consistent with this, rice accumulated substantial soluble sugars under low-temperature stress, a response that correlated with enhanced cold tolerance. Notably, KY131 displayed a distinct sugar metabolic shift. This metabolic signature aligned closely with the reported pollen cold-susceptible pathway: cold stress suppressed anther cell wall invertase *OsINV4*, trapping sucrose inside anthers and cutting hexose delivery to developing pollen, which triggered pollen sterility [9]. Notably, F6P accumulation in KY131 young panicles and was positively correlated with SOD and CAT activities, but as a steady-state measure, does not prove enhanced PPP flux or NADPH production [58].

### 4.4. The Candidate OsbZIP69–OsHXK7 Association and the Cold-Tolerance Mechanism in Cold-Tolerant Rice

Integrated transcriptomic and WGCNA analyses identified 692 core DEGs and 16 co-expression modules, with intersection analysis revealing three overlapping hub TFs. Among these, the bZIP transcription factor *OsbZIP69* exhibited the highest connectivity and showed strongest co-expression with and transcriptional targeting of the hexokinase gene *OsHXK7*. The hub genes were significantly enriched in sugar metabolism pathways (e.g., starch/sucrose and amino/nucleotide sugar metabolism), consistent with metabolomic evidence of carbohydrate accumulation (i.e., F6P). Furthermore, bZIP TFs are well-established regulators of plant abiotic stress tolerance [29,30,59]. For instance, *OsbZIP72* enhanced rice cold tolerance at both seedling and booting stages by activating ROS-scavenging genes to sustain ROS homeostasis under low temperature [30], while *OsbZIP62* improved drought and oxidative stress resistance [59]. Gene annotation further suggested that *OsbZIP69* (Os08g0549600) participated in flowering regulation, supporting its potential functional relevance to reproductive-stage cold tolerance [35].

The dual biological roles of hexokinase formed the mechanistic foundation of the *OsbZIP69*-*OsHXK7* regulatory module. As the rate-limiting glycolytic enzyme, HXK phosphorylated glucose and fructose to generate G6P and F6P. These catalytic products supplied carbon skeletons for energy metabolism and osmoprotection under cold stress, and fueled NADPH production through the pentose phosphate pathway to support antioxidant machinery [31,32,33]. Separately, HXK operated as an intracellular glucose sensor that bridged sugar signaling and phytohormone regulatory cascades [33,34]. Nevertheless, overactivated glucose-sensing activity triggered detrimental oxidative damage. Wheat experiments showed that overexpressing *TaHXK7-1A* increased glucose sensitivity, upregulated RBOH genes, and drove excessive O_2_^−^ and MDA buildup, which compromised drought tolerance in transgenic *Arabidopsis* and wheat [35]. Parallel results were documented in rice: HXK overexpression triggered ROS bursts, and ectopic *OsHXK1* expression upregulated *OsRBOH* and accelerated leaf senescence [60,61]. These consistencies implied that the TF-*HXK7*-ROS homeostasis module represented a conserved functional unit governing stress resistance across cereal crops, advancing our current understanding of cold adaptation in northern japonica rice.

### 4.5. Limitations

Several limitations of this study should be acknowledged. (1) A single cold-tolerant and a single cold-sensitive cultivar were compared, which limits the generalizability of the conclusions across the japonica gene pool. (2) The multi-omics integration is correlational and does not establish causality. (3) The hypothesized *OsbZIP69*-*OsHXK7* regulatory relationship lacks functional validation (Y1H, EMSA, dual-luciferase, and transgenic assays). (4) Metabolomics relied on a single LC-MS/MS platform, which constrains metabolite coverage. Future work will address these limitations through functional validation in larger germplasm panels.

## 5. Conclusions

This study systematically characterized differential responses in agronomic traits, antioxidant physiology, metabolome, and transcriptome between two rice cultivars under booting-stage cold stress, and identified core cold-tolerance genes through integrated WGCNA. The results showed that (1) the KY131 retained significantly higher SSR and GW; (2) KY131 mitigated O_2_^−^ and H_2_O_2_ accumulation by sustaining elevated SOD, POD, and CAT activities, with multivariate analyses establishing SOD activity as the foremost physiological discriminant; (3) metabolomics pinpointed F6P as the core differential metabolite among 101 identified DAMs; and (4) transcriptomics identified 692 DEGs, leading to the identification of the candidate hub TF *OsbZIP69*, which showed the strongest co-expression association with the glucose sensor gene *OsHXK7*. These results identify *OsbZIP69* and *OsHXK7* as priority candidate genes for the functional dissection of booting-stage cold tolerance in cold-region japonica rice, and reveal an association between sugar-phosphate metabolism (centered on F6P) and redox homeostasis that generates a testable hypothesis for future studies.

## Figures and Tables

**Figure 1:**
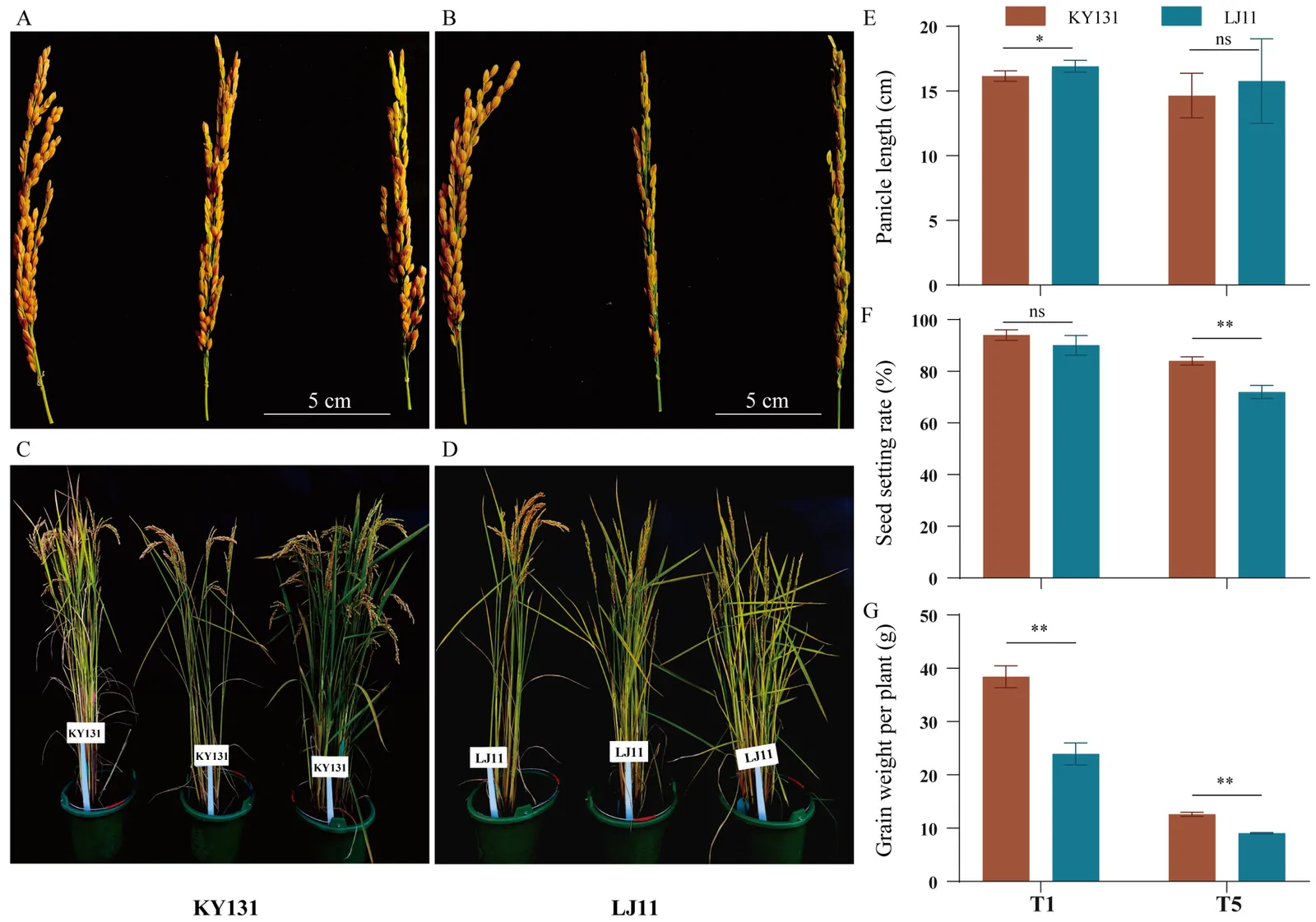
**Effects of cold stress at the booting stage on rice agronomic traits.** (**A**,**B**) Panicle phenotypes at maturity after 0 h (T1) and 120 h (T5) of cold treatment at the booting stage. (**C**,**D**) Appearance of KY131 and LJ11 plants at maturity after cold treatment at the booting stage. (**E**–**G**) Panicle length, seed setting rate, and grain weight per plant at maturity after 0 h and 120 h of cold treatment (*, *p* < 0.05; **, *p* < 0.01; ns, *p* > 0.05).

**Figure 2:**
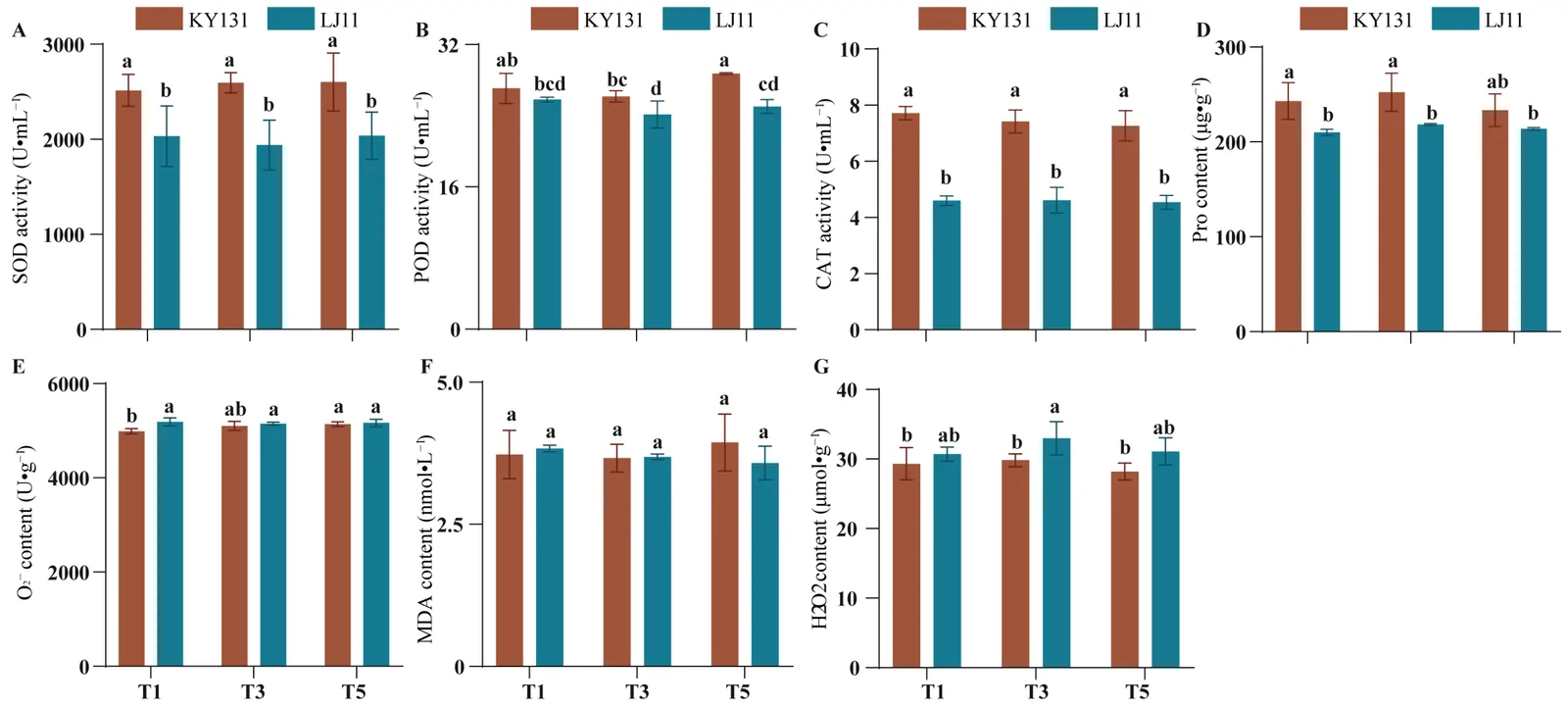
**Antioxidant enzyme activities and oxidative stress marker contents in young rice panicles under cold stress.** (**A**–**C**) SOD, POD, and CAT activities in young panicles at 0, 72, and 120 h of cold treatment. (**D**–**G**) Pro, O_2_^−^, MDA, and H_2_O_2_ contents in young panicles under cold stress. Different lowercase letters above the bars indicate significant differences (*p* < 0.05) by LSD test; capped bars represent the standard error of the mean (SEM).

**Figure 3:**
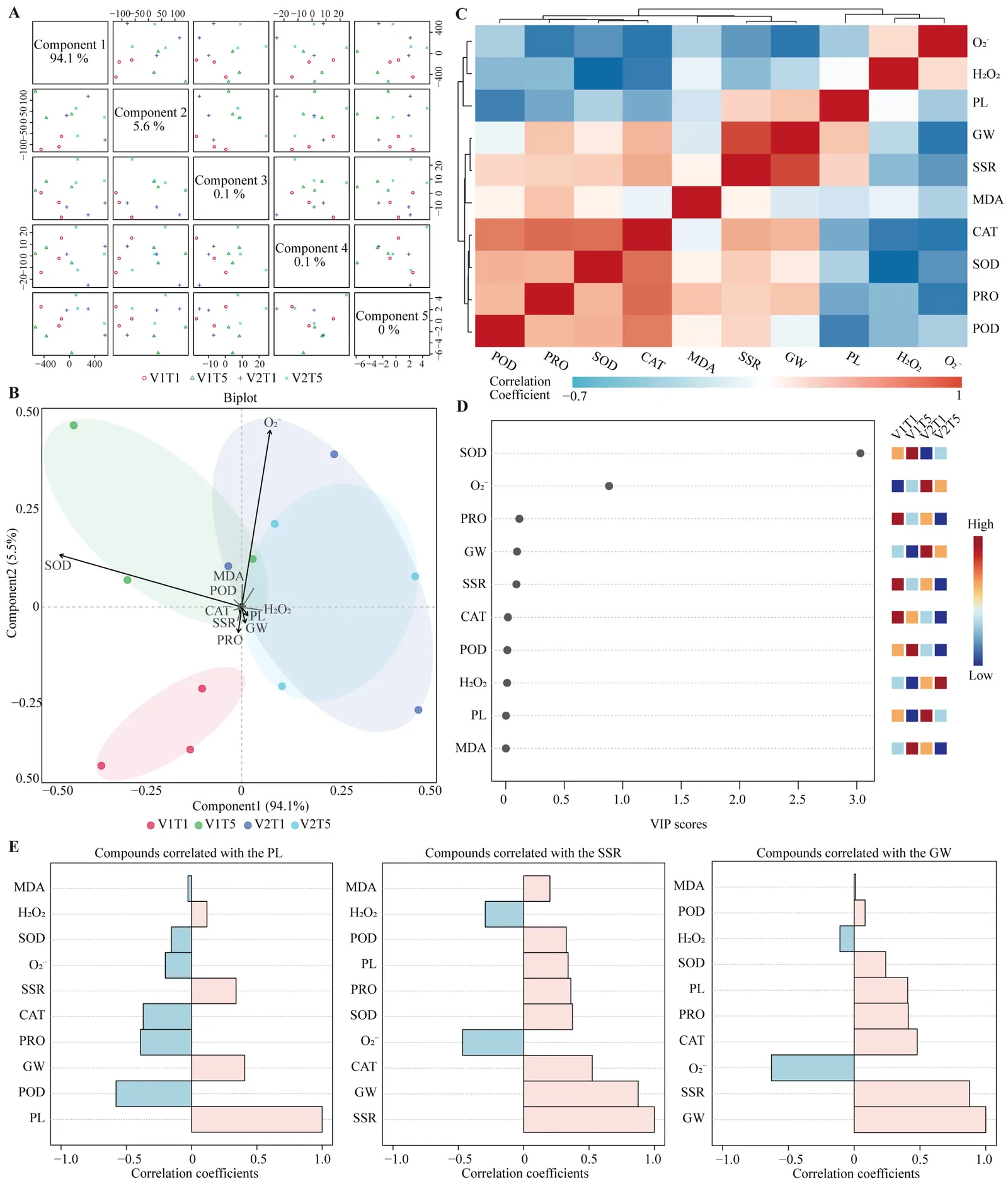
**Multivariate statistical analysis of agronomic traits and antioxidant indices of the two varieties under cold treatment.** (**A**) PCA scatter matrix of the ten indices across four treatment groups (V1T1, V1T5, V2T1, V2T5). V1 denotes cold-tolerant KY131; V2 denotes cold-sensitive LJ11; T1 and T5 denote 0 and 120 h of 15 °C cold treatment, respectively. (**B**) PCA biplot arrows indicated loading directions of each index. (**C**) Pearson correlation heatmap among the ten indices. (**D**) VIP plot and content heatmap of each index across the four treatment groups (red = high, blue = low). (**E**) Bar charts of correlation coefficients between each index and panicle length (PL), seed setting rate (SSR), and grain weight per plant (GW).

**Figure 4:**
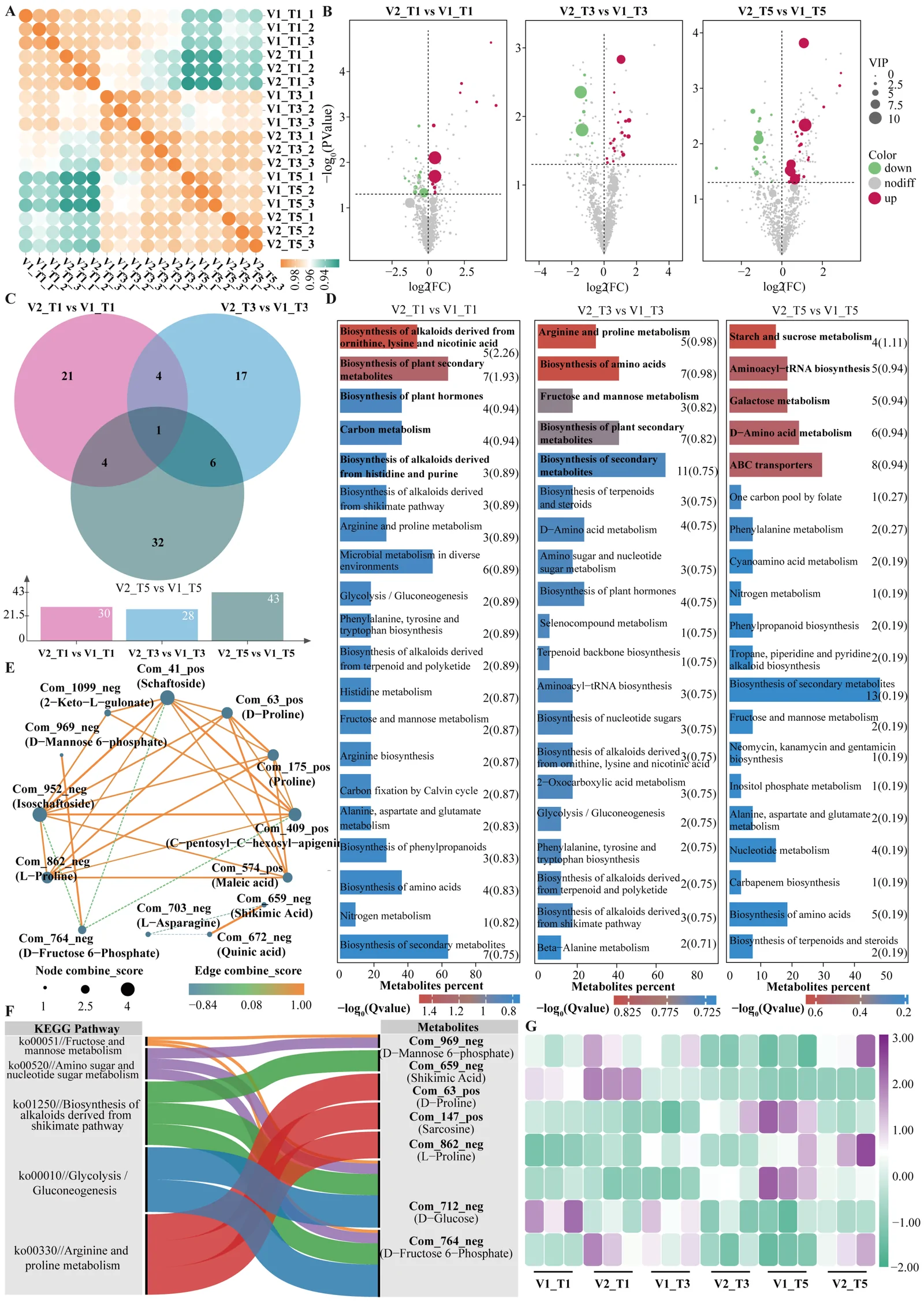
**Effects of cold stress at the booting stage on the rice metabolome.** (**A**) Correlation heatmap of metabolites among samples of the two varieties at different time points. (**B**) Volcano plots of DAMs in the three comparison groups (V2_T1 vs. V1_T1, V2_T3 vs. V1_T3, V2_T5 vs. V1_T5). (**C**) Venn diagram and statistics of DAMs among the three comparison groups. (**D**) KEGG enrichment pathways of DAMs in each comparison group. (**E**) Correlation network of the 13 overlapping DAMs (node size indicates connectivity; orange lines = positive correlation, green dashed lines = negative correlation). (**F**) Sankey diagram of core pathways and the seven core metabolites. (**G**) Content heatmap of the seven core metabolites across the six treatment groups.

**Figure 5:**
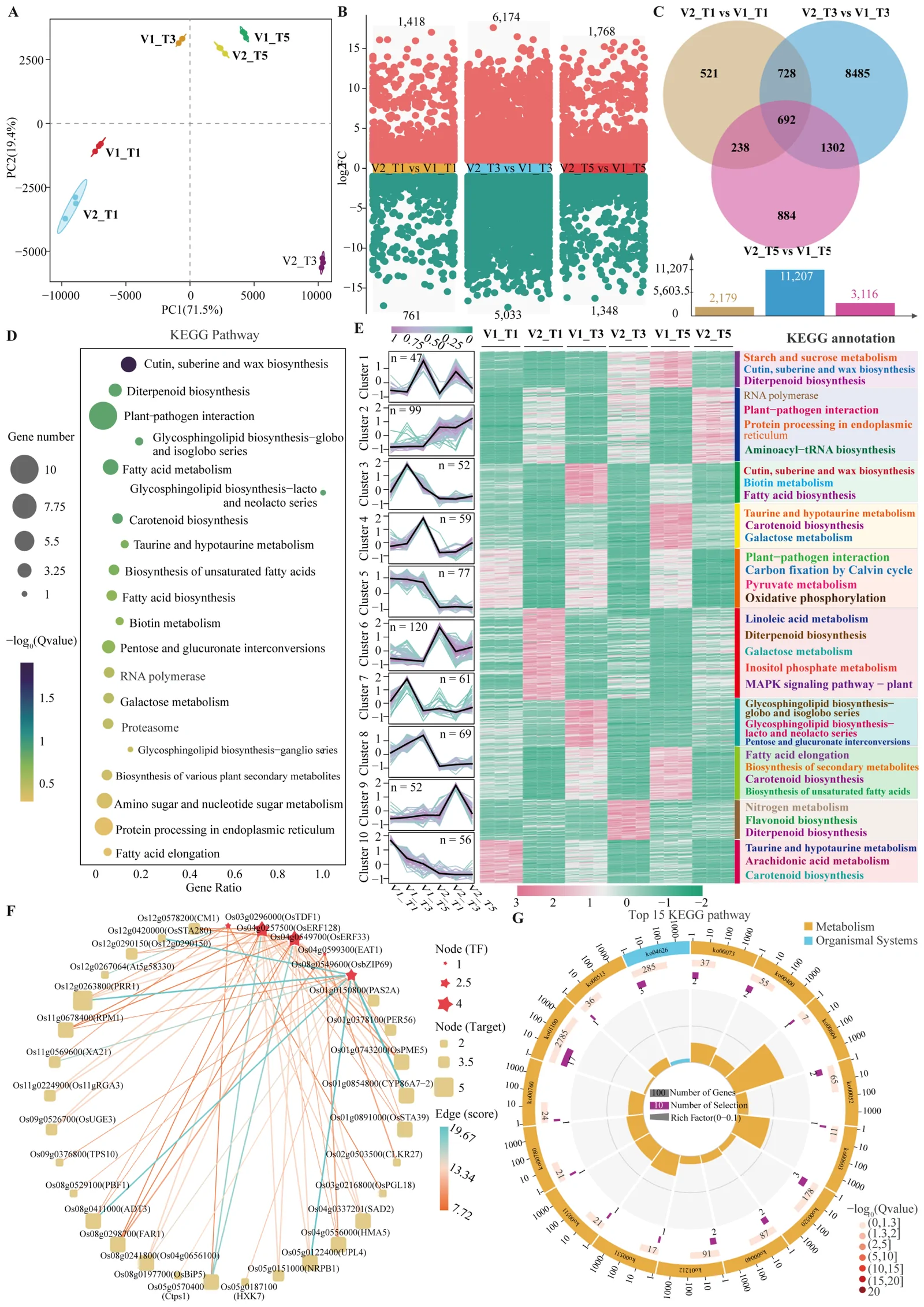
**Transcriptomic landscapes in the cold-tolerant cultivar KY131 and the cold-sensitive cultivar LJ11 under cold stress.** (**A**) PCA of all gene transcripts of the two varieties at different time points. (**B**) Statistics of up- and down-regulated DEGs in the three comparison groups. Different colours represent different comparison groups. (**C**) Venn diagram of common and specific DEGs among the three comparison groups. (**D**) KEGG enrichment bubble plot of the 692 common DEGs. (**E**) Mfuzz clustering, expression heatmap, and KEGG annotation of each cluster for the 692 common DEGs. (**F**) TF-target gene co-expression network (red stars = TFs; yellow squares = target genes). (**G**) KEGG circular enrichment plot of the core network genes (Top 15).

**Figure 6:**
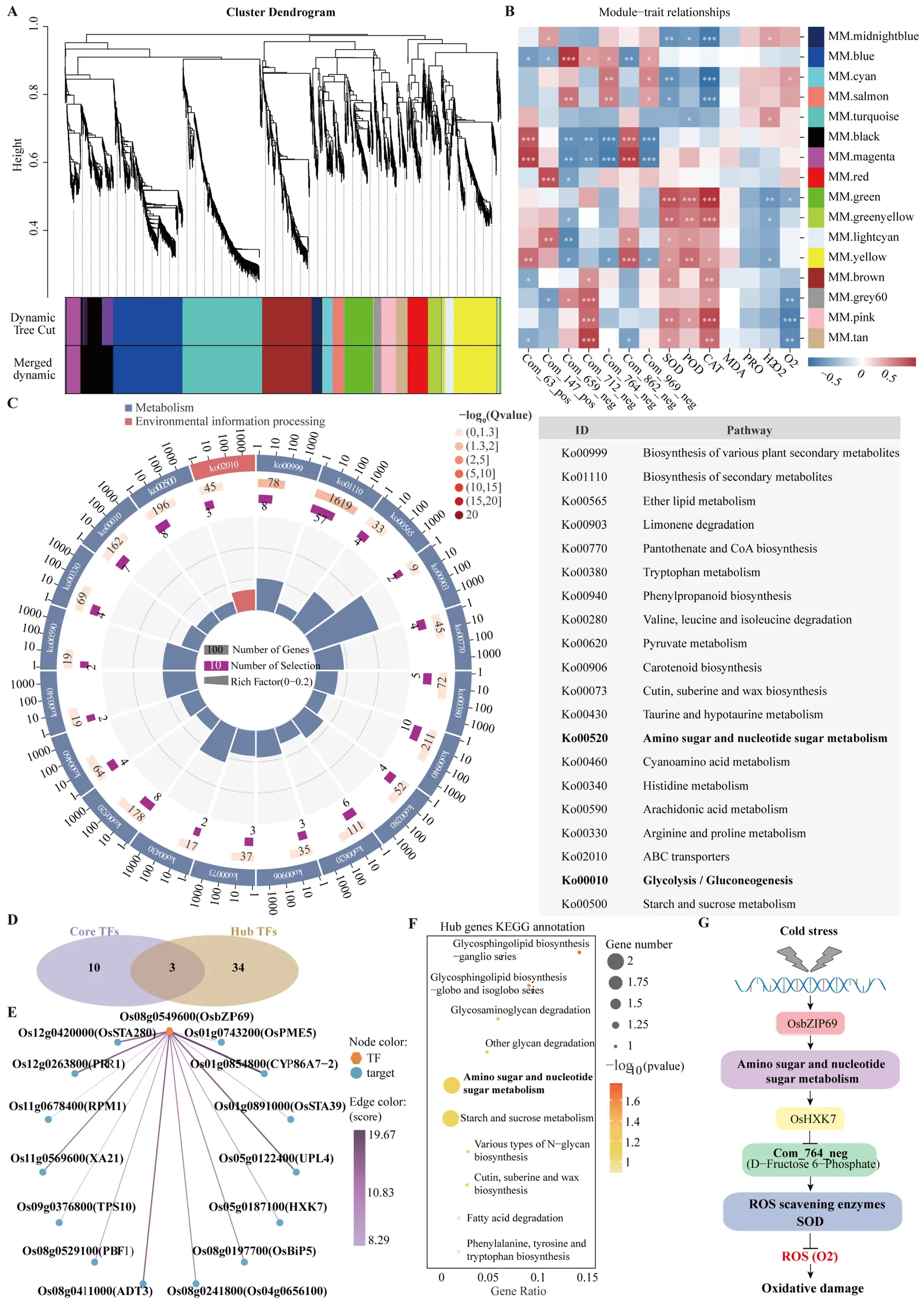
**Integrated WGCNA results and the cold-tolerance regulatory model.** (**A**) Cluster dendrogram of gene co-expression modules; major branches form 16 modules marked with different colors. (**B**) Module-trait correlation heatmap; each row represents a module and each column a metabolite/physiological index (*, **, and *** indicate *p* < 0.05, 0.01, and 0.001, respectively). (**C**) KEGG circular enrichment plot of genes in modules significantly correlated with core DAMs. (**D**) Venn diagram of core TFs and module hub TFs (37). (**E**) Co-expression network of the hub transcription factor *OsbZIP69* and its target genes (**F**) KEGG enrichment bubble plot of hub genes. (**G**) A hypothetical working model of the putative *OsbZIP69*-*OsHXK7* association with F6P accumulation and antioxidant homeostasis under booting-stage cold stress.

## Data Availability

The raw data of RNA-seq were deposited in the Genome Sequence Archive (GSA) of the National Genomics Data Center (NGDC) (Accession no. PRJCA070124, https://ngdc.cncb.ac.cn/gsa/search?searchTerm=CRA049622, accessed on 15 September 2026). The other relevant data are presented in this paper or Supplemental Materials.

## Supplementary Materials

The following supporting information can be downloaded at: https://www.mdpi.com/article/10.3390/antiox15101245/s1, Table S1: Two-way ANOVA of agronomic traits and antioxidant indices with cultivar, cold duration, and their interaction as fixed effects; Table S2: The differentially expressed metabolites in V2_T1-vs.-V1_T1,V2_T3-vs.-V1_T3, and V2_T5-vs.-V1_T5; Table S3: The core differential metabolites between groups; Table S4: Overview of RNA-seq data; Table S5: The common DEGs among groups; Table S6: The upregulated DEGs of V2_T1-vs.-V1_T1, V2_T3-vs.-V1_T3, and V2_T5-vs.-V1_T5 in the three comparison groups; Table S7: The TFs and target gene prediction analysis; Table S8: The DEGs were used for WGCNA; Table S9: The DEGs significantly associated with DEMs in module-trait associations; Table S10: The hub TFs and target gene prediction analysis.
